# Evidence of Pyrimethamine and Cycloguanil Analogues as Dual Inhibitors of *Trypanosoma brucei* Pteridine Reductase and Dihydrofolate Reductase

**DOI:** 10.3390/ph14070636

**Published:** 2021-06-30

**Authors:** Giusy Tassone, Giacomo Landi, Pasquale Linciano, Valeria Francesconi, Michele Tonelli, Lorenzo Tagliazucchi, Maria Paola Costi, Stefano Mangani, Cecilia Pozzi

**Affiliations:** 1 Department of Biotechnology, Chemistry and Pharmacy, Department of Excellence 2018–2022, University of Siena, via Aldo Moro 2, 53100 Siena, Italy; giusy.tassone@unisi.it (G.T.); landi31@unisi.it (G.L.); stefano.mangani@unisi.it (S.M.); 2 Department of Life Science, University of Modena and Reggio Emilia, via Campi 103, 41125 Modena, Italy; pasquale.linciano@unipv.it (P.L.); lorenzo.tagliazucchi@unimore.it (L.T.); mariapaola.costi@unimore.it (M.P.C.); 3 Department of Pharmacy, University of Genoa, Viale Benedetto XV n.3, 16132 Genoa, Italy; francesconi.phd@difar.unige.it (V.F.); tonelli@difar.unige.it (M.T.)

**Keywords:** pyrimethamine, cycloguanil, derivatives, *Trypanosoma brucei*, pteridine reductase, dihydrofolate reductase, antifolate drug, dual inhibitor, X-ray crystallography, structure–activity relationship

## Abstract

*Trypanosoma* and *Leishmania* parasites are the etiological agents of various threatening neglected tropical diseases (NTDs), including human African trypanosomiasis (HAT), Chagas disease, and various types of leishmaniasis. Recently, meaningful progresses in the treatment of HAT, due to *Trypanosoma brucei* (*Tb*), have been achieved by the introduction of fexinidazole and the combination therapy eflornithine–nifurtimox. Nevertheless, due to drug resistance issues and the exitance of animal reservoirs, the development of new NTD treatments is still required. For this purpose, we explored the combined targeting of two key folate enzymes, dihydrofolate reductase (DHFR) and pteridine reductase 1 (PTR1). We formerly showed that the *Tb*DHFR inhibitor cycloguanil (CYC) also targets *Tb*PTR1, although with reduced affinity. Here, we explored a small library of CYC analogues to understand how their substitution pattern affects the inhibition of both *Tb*PTR1 and *Tb*DHFR. Some novel structural features responsible for an improved, but preferential, ability of CYC analogues to target *Tb*PTR1 were disclosed. Furthermore, we showed that the known drug pyrimethamine (PYR) effectively targets both enzymes, also unveiling its binding mode to *Tb*PTR1. The structural comparison between PYR and CYC binding modes to *Tb*PTR1 and *Tb*DHFR provided key insights for the future design of dual inhibitors for HAT therapy.

## 1. Introduction

Protozoan parasites belonging to the *Trypanosoma* and *Leishmania* species are the etiological agents of various threatening neglected tropical diseases (NTDs), including human African trypanosomiasis (HAT, also known as sleeping sickness), Chagas disease, and different forms of leishmaniasis [1,2]. HAT and Chagas disease are caused by *Trypanosoma brucei* (*T. brucei* or *Tb*) and *Trypanosoma cruzi* (*T. cruzi*) infection, respectively, while *Leishmania* spp. cause various types of leishmaniasis, spanning from cutaneous lesions to potentially fatal visceral forms [3]. NTDs are endemic in many countries; the World Health Organization (WHO) has estimated that billions of people worldwide are at risk of these parasite infections and millions of clinical cases are reported every year [4]. The current therapeutic regimens against these parasite diseases are limited and burdened by high toxicity, poor efficacy, and rapid insurgence of resistance [5]. In recent years, important steps forward in the treatment of HAT have been made by the introduction of the combination therapy eflornithine–nifurtimox and of fexinidazole, the first oral drug against this disease [6,7,8,9]. Despite the efforts to control HAT, drug resistance issues may still occur—a problem further exacerbated by the exitance of animal reservoirs of these diseases [10,11,12]. Thus, the development of new drugs effective against protozoan parasites is still an urgent need.

**Figure 1:**
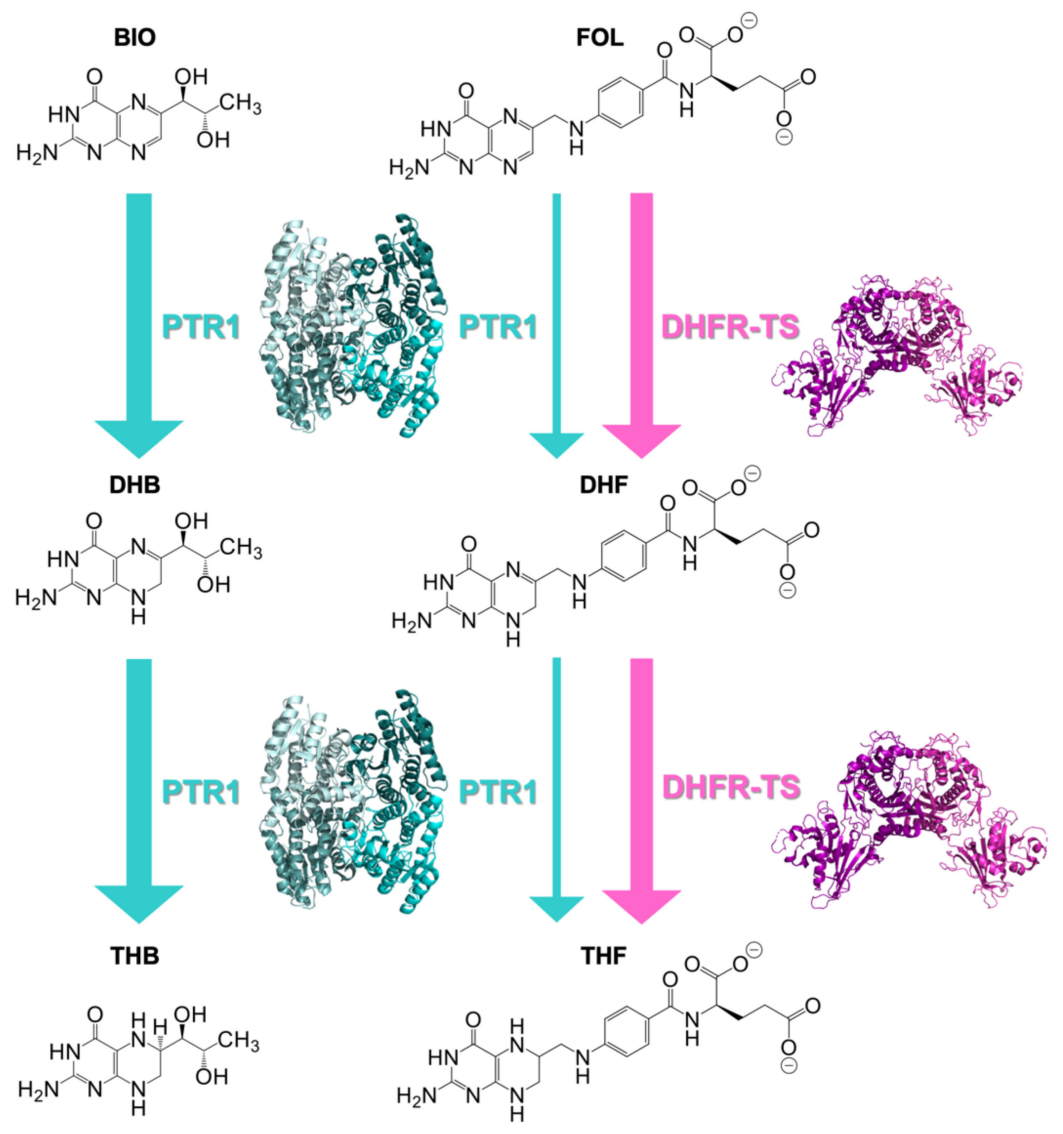
Two-stage reduction of biopterin (BIO) to 7,8-dihydrobiopterin (DHB) and 5,6,7,8-tetrahydrobiopterin (THB) catalyzed by PTR1 and of folate (FOL) to 7,8-dihydrofolate (DHF) and 5,6,7,8-tetrahydrofolate (THF) catalyzed by both dihydrofolate reductase-thymidylate synthase (DHFR-TS) and PTR1. Enzymes are shown next to the reactions that they catalyze. The thicker arrows indicate that the enzyme can contribute to the metabolic step only under specific metabolic conditions. Indeed, DHFR-TS is mainly responsible for the folate-reduction pathway, whereas PTR1 accounts for the biopterin one, being involved in the folate-reduction subsequently to DHFR inhibition.

Targeting the enzymes of the folate metabolism was proven as a successful strategy against bacterial infections [13] and malaria [14], and, more recently, it has been exploited also for the development of novel antiparasitic treatments [15,16,17,18,19,20,21,22,23,24]. Trypanosomes are unable to synthesize folates and pterins required for critical cellular metabolic pathways, such as the biosynthesis of nucleic acids and proteins [25]. To survive, trypanosomes retrieve extracellular pterin precursors from their hosts [26,27]. Following the uptake, pterins (biopterin or folate) undergo two successive reductions to yield the active tetrahydro-derivatives (Figure 1) [26]. In trypanosomes, the key enzymes involved in the provision of these reduced molecular species are dihydrofolate reductase (DHFR, EC 1.5.1.33) and pteridine reductase 1 (PTR1, EC 1.5.1.33) [28,29,30]. DHFR is a reduced nicotinamide adenine dinucleotide phosphate (NADPH)-dependent enzyme that catalyzes the two-step reduction of folate to 7,8-dihydrofolate (DHF) and subsequently to 5,6,7,8-tetrahydrofolate (THF) (Figure 1) [31,32,33]. In trypanosome parasites, this is a domain of the bifunctional enzyme DHFR-thymidylate synthase (DHFR-TS). However, traditional antifolates, such as methotrexate (MTX, Figure S1, Supplementary Materials) inhibiting DHFR, are poorly effective towards trypanosome parasites because of the metabolic bypass provided by PTR1, a NADPH-dependent enzyme belonging to the short-chain dehydrogenase/reductase (SDR) family [26,29]. In addition to folate reduction, PTR1 also catalyzes the conversion of biopterin to 7,8-dihydrobiopterin (DHB) and subsequently to 5,6,7,8-tetrahydrobiopterin (THB) (Figure 1) [31,32,33]. Under DHFR inhibition, the gene is upregulated, providing reduced folates necessary for parasite survival [26,31,34,35]. Furthermore, *PTR1* gene knockdown and knockout studies in *T. brucei* have proven that parasite growth is impaired both in vitro and in the animal host [29,36]. This evidence supports that the concomitant inhibition of both DHFR and PTR1 enzyme activities represents a successful strategy to develop novel HAT treatments.

**Figure 2:**
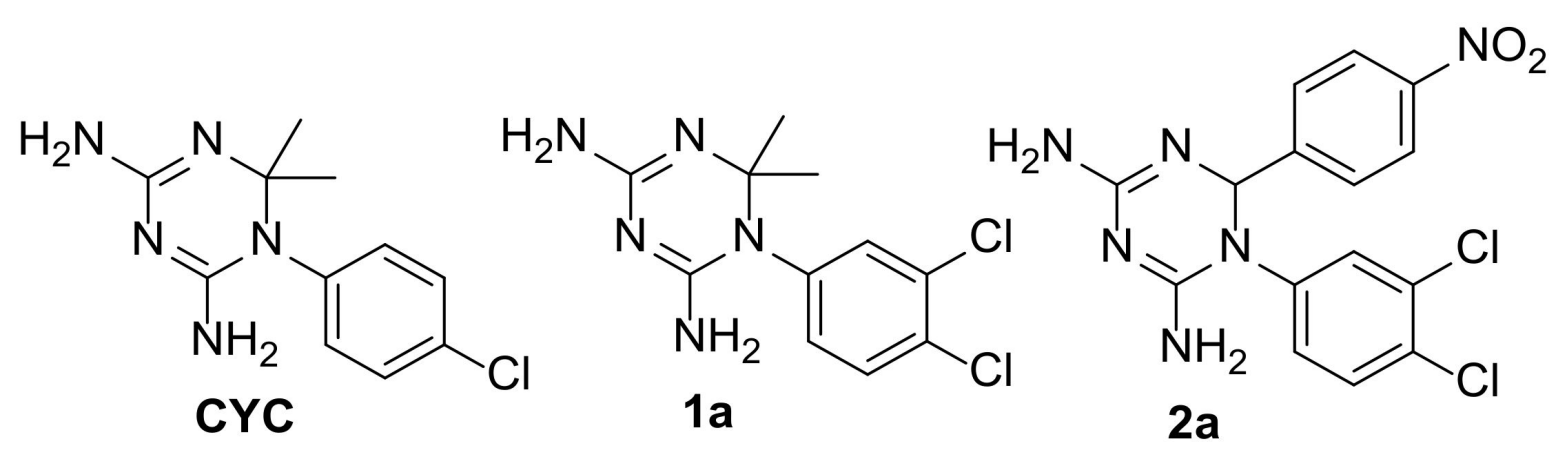
CYC and its analogues previously studied as *Tb*PTR1 inhibitors [20].

To date, a wide variety of inhibitors has been developed for targeting both folate enzymes, but only a few are able to provide efficacy in vitro, and it is still difficult to obtain inhibitors of both enzymes with a similar inhibition potency. In this context, we recently reported the experimental evidence of cycloguanil (CYC), a known inhibitor of *Plasmodial* and *Trypanosomal* DHFR-TS enzymes, also as an inhibitor of *Tb*PTR1 [20]. The structural characterization of *Tb*PTR1 in complex with this dihydrotriazine derivative disclosed the binding features of this modest inhibitor and guided the design of a small set of analogues (Figure 2). CYC showed an IC_50_ of 31.6 μM towards *Tb*PTR1, while its best performing derivatives (**1a** and **2a**) were characterized by the improvement of the IC_50_ up to 200 times as a result of the introduction of the 3,4-diCl substitution on the phenyl ring in position 5 of dihydrotriazine (**1a**, IC_50_ = 0.692 μM) and by the further replacement of the dimethyl group on the dihydrotriazine C(6) with a 4-nitrophenyl ring (**2a**, IC_50_ = 0.186 μM). This higher potency was rationalized by comparing the X-ray crystal structure of the *Tb*PTR1:NADP(H):CYC complex (PDB id 6HNC) with those obtained with the derivatives **1a** (PDB id 6HNR) and **2a** (PDB id 6HOW) [20].

The pteridine and pyrimidine core structures have largely been explored with the intent to discover new anti-trypanosomatidic drugs, also taking advantage of the information derived from the available crystal structures, which allowed the further decoration of their scaffolds [15,16,17,18,19,20,21,22,23,24]. Pursuing our interest in the search of more effective agents against the *T. brucei* parasite, we deemed worthwhile the study of the anti-trypanosomatidic activity of two known drugs, pyrimethamine (PYR) and MTX (Figure S1, Supplementary Materials). Notably, MTX and PYR showed IC_50_ values in the nanomolar level for both *Tb*PTR1 and *Tb*DHFR [37,38] (Table 1). For both drugs, the structural basis of their inhibition potency against the parasitic enzymes was studied, and the X-ray crystal structures are available for *Tb*PTR1 in complex with MTX (*Tb*PTR1:NADP(H):MTX, PDB id 2C7V [22]) and for *Tb*DHFR (isolated domain) in complex with PYR (*Tb*DHFR:NADP(H):PYR, PDB id 3QFX [37]). PYR is known to target *Tb*DHFR and has an inhibition constant (*K_i_*) of 24.2 nM [37]. Thus, we provided experimental and structural evidence of the additional ability of PYR as a *Tb*PTR1 inhibitor.

The dual inhibition of the enzymes by PYR (IC_50_ *Tb*PTR1 = 90 nM and IC_50_ *Tb*DHFR = 45 nM) is an interesting finding and structural analysis of its binding mode may suggest the main requirements to ensure such an ability [39].

Compared with PYR, the potency of CYC towards *Tb*PTR1 was surprisingly at least three orders of magnitude lower (See Table 1). It is interesting to understand how PYR and CYC, despite their substantial structural similarity, show a significantly different inhibition potency. Accordingly, in the present work, we analyzed the structural basis of CYC versus PYR inhibition of *Tb*PTR1 and then explored some CYC analogues (**1**-**6**) with a modified substitution pattern to understand how targeted structural modification of CYC can influence the inhibition ability towards both *Tb*PTR1 and *Tb*DHFR [20,22,35]. The structural comparison between the binding mode of PYR and CYC in *Tb*PTR1 and *Tb*DHFR led to key insights for the future design of more promising dual inhibitors for HAT therapy. Meanwhile, attaining the goal of potency and selectivity for parasitic DHFR with respect to the human isoform (hDHFR) continues to be a challenge that affects the design of new antifolates [40,41]. This is due to the evolutionary conservation of the *DHFR* gene, although subtle differences in the active sites of human and protozoan enzymes have been pointed out [42]. Non-selective DHFR inhibitors, such as MTX, may cause dose-limiting toxicities, requiring expensive concomitant folate rescue therapy (e.g., leucovorin), combination regimens with other drugs or discontinuation of the treatment. Therefore, we investigated the species selectivity profile of our compounds, analyzing the main chemical features responsible for their preferential activity towards the protozoan enzyme.

## 2. Results

### 2.1. Structural Characterization of the TbPTR1:NADP(H):PYR Complex

**Figure 3:**
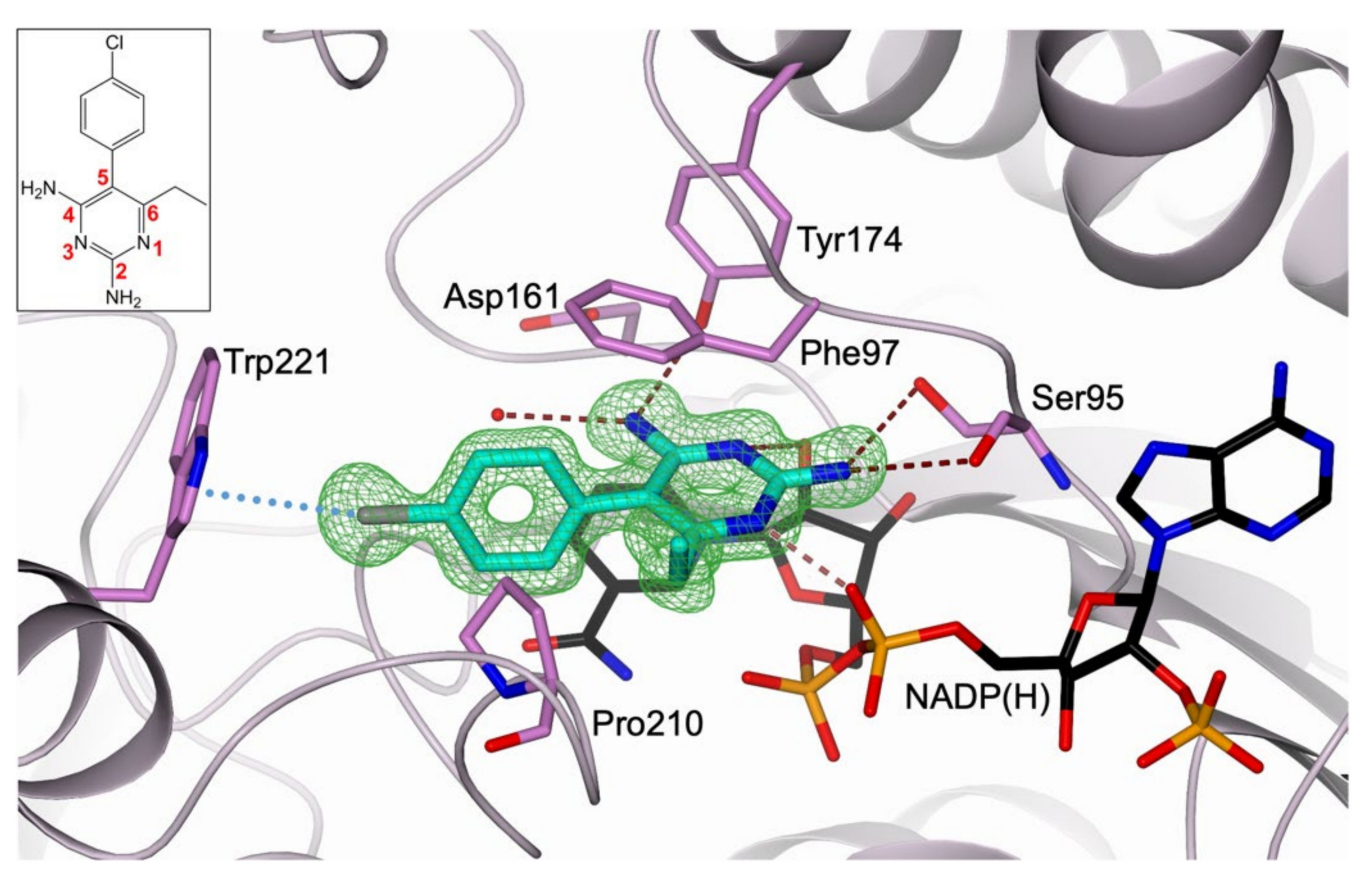
Active-site view of *Tb*PTR1 (lilac cartoon; residues in sticks, orchid carbon atoms) in complex with NADP(H) (in sticks, black carbon atoms) and PYR (in sticks, cyan carbon atoms). PYR is surrounded by the omit map (green mesh) contoured at the 3.0 σ level. The PYR chemical structure and its atom numbering are shown in the inset. Water molecule is displayed as a red sphere. In all figures, hydrogen and halogen bonds are represented as tan dashed lines and light-blue dotted lines, respectively. Oxygen atoms are colored red, nitrogen blue, phosphorus orange, and chlorine grey.

The structure of *Tb*PTR1 in complex with the cofactor NADP(H) and PYR was obtained with a 1.34 Å resolution, providing a detailed view of the inhibitor interactions within the enzyme catalytic cavity (Table S1, Supplementary Materials). The crystal asymmetric unit includes a whole functional *Tb*PTR1 tetramer, whose structure is highly conserved compared with those previously described (subunits were completely traced apart for two surface-exposed loops including residues 103–113 and 143–151, usually poorly organized in *Tb*PTR1 structures) [15,16,17,21,23]. The active site is an L-shaped depression mainly built within a single protein chain having one end blocked by the C-terminus of a partner subunit. Inside this cavity, NADP(H) binds in an extended conformation entrapped by a tight network of conserved H-bonds [22,25]. The binding of the cofactor is essential for creating both the catalytic site and the substrate-binding pocket [23,36], where PYR binds through the peculiar π-sandwich between the cofactor nicotinamide and the aromatic side chain of Phe97 [25,36]. The PYR amine moiety in position 2 (atom numbering in the inset of Figure 3) donates H-bonds to the Ser95 hydroxyl and backbone carbonyl groups (Figure 3). The nitrogen atoms in positions 1 and 3 directly interact with the cofactor, forming H-bonds with its ribose hydroxyl and β-phosphate groups, respectively. The amine moiety at C(4) donates a H-bond to the Tyr174 hydroxyl group, that is further H-bonded to the Asp161 carboxylate, a key interaction within the *Tb*PTR1 catalytic cavity, playing a prominent role during catalysis [22]. The ethyl moiety in position 6 forms van der Waals interactions with both Phe97 and Pro210. In the binding mode adopted by PYR, the 1-(4-chlorophenyl) ring is rotated by 74° with respect to the 2,4-diamine-6-ethylpyrimidine ring. This rotation places the 4-Cl atom in an optimal position to establish a halogen bond with the indole moiety of Trp221, pointing exactly to the centre of its five-membered ring. PYR fully populates the biopterin-binding pocket in three subunits of the tetramer (chains A, B, and D of our model) whereas a reduced occupancy (60%) is estimated in the fourth subunit (chain C), in which the population of the cofactor is also reduced (60%, consistently with PYR). A correlation between the decreased population of the cofactor site and the increased flexibility of the substrate-binding loop (including residues 207–215) was previously reported by us [15,16]. Indeed, in subunit C, residues 206–222 were refined to a 60% occupancy (consistently with the cofactor and PYR) and, among these, residues 212–216 resulted in highly flexible loops and were excluded from the model.

### 2.2. Selection of the Small Library of CYC Derivatives

The modest inhibition of *Tb*PTR1 shown by CYC (IC_50_ = 31.6 µM) prompted us to select new analogues to discover more effective inhibitors that may show a dual inhibitory activity against *Tb*PTR1 and *Tb*DHFR.

**Figure 4:**
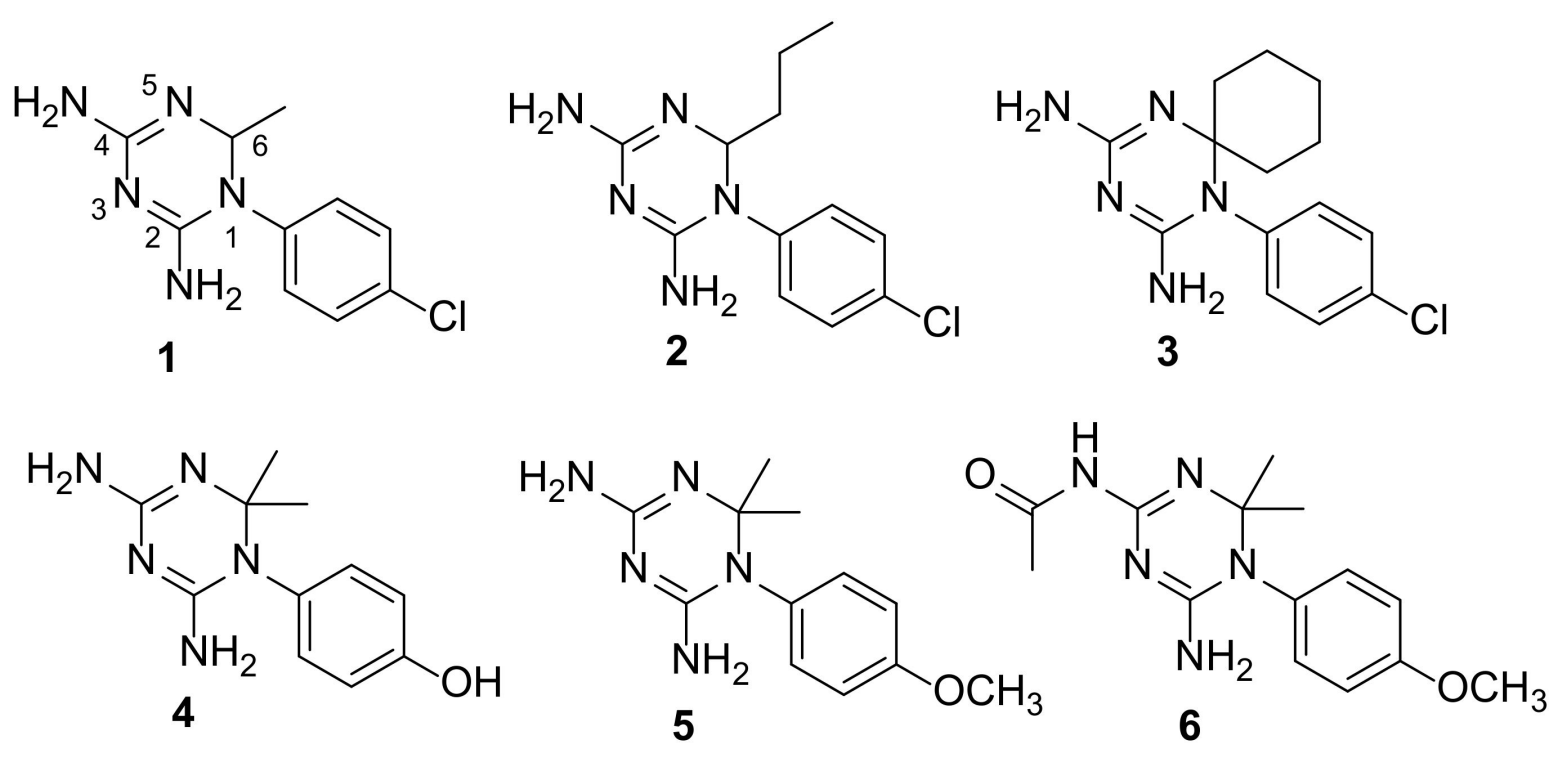
Chemical structures of CYC analogues **1–6**.

The selected CYC analogues (**1–5**, Figure 4) have previously demonstrated to inhibit the influenza virus and respiratory syncytial virus (RSV) replication, dampening the activity of the host factor (cellular) DHFR. They proved efficacy in the low micromolar range, without producing manifest toxicity (CC_50_ > 100 µM) against four animal and human cell lines, also showing intracellular target specificity [38]. These compounds have structural features that we considered interesting to be explored against the parasitic enzymes. On the basis of visual inspection of the three X-ray structures—*Tb*PTR1:NADP(H):PYR (PDB id 7OPJ, present work) and *Tb*PTR1:NADP(H):CYC (PDB id 6HNC [20]) and *Tb*DHFR (*Tb*DHFR:NADP(H):PYR, PDB id 3QFX [37])—we found that the chemical variation of the 4-Cl substituent on the phenyl ring and/or of the two methyl groups at C(6) of CYC could improve the compound affinity. Additionally, we have also obtained the acetamido derivative of compound **5** to increase the compound lipophilicity and the bulkiness of the substituent out of the amino group at C(4).

### 2.3. Synthesis of CYC Derivatives

We deemed interesting to synthesize and evaluate the *Tb*PTR1 and *Tb*DHFR inhibitory activities of a set of CYC analogues bearing in position 1 an aromatic ring, variously substituted, and in position 6 one or two alkyl moieties (Figure 4).

**Scheme 1:**
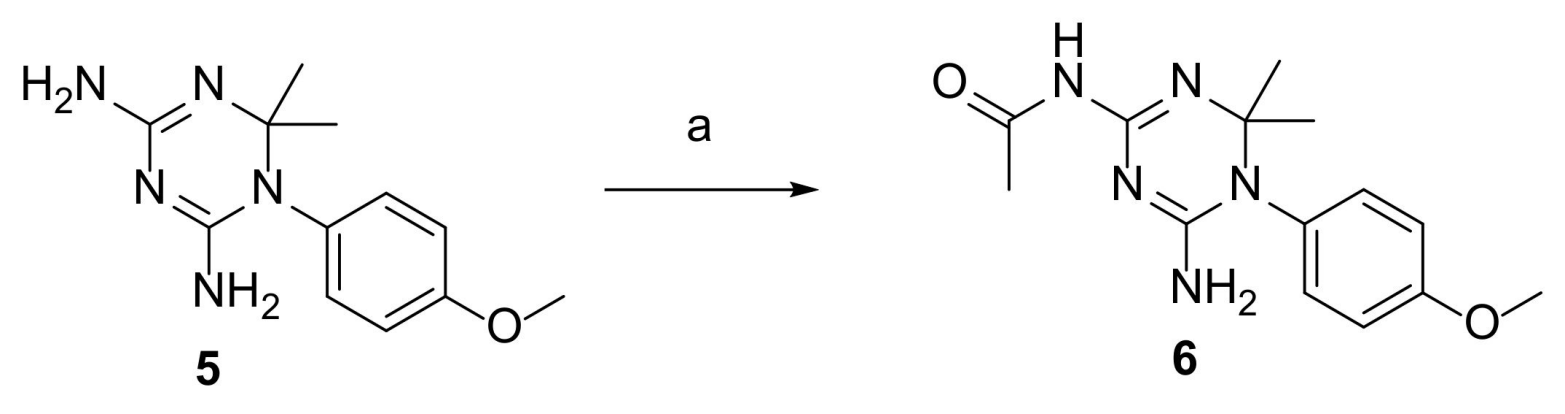
Reagents and conditions: a—Ac_2_O (3 equivalents), CH_3_OH, room temperature, 2 h.

The dihydrotriazines **1–5** were synthesized according to cited references: compounds **1** and **2** were obtained by means of the two-component syntheses involving the preformed 1-(4-chlorophenyl)biguanide and an aldehyde derivative [43], while for **3** [44], **4** [45], and **5** [46], the three-component synthetic method of Modest was applied by reacting the appropriate *para*-substituted aniline hydrochloride salt, dicyandiamide, and acetone. The treatment of 4,6-diamino-1-(4-methoxyphenyl)-2,2-dimethyl-1,2-dihydrotriazine (**5**) with an excess of acetic anhydride at room temperature for 2 h yielded the corresponding N-acetyl derivative (**6**) (Scheme 1).

The structure of the novel compound was confirmed using ^1^H and ^13^C NMR and elemental analysis. On the basis of the NMR spectra, only the N-acetyl derivative (**6**) formed in the reaction, which most probably occurred on the more accessible NH_2_ group at C(4) of dihydrotriazine **5**; the reactivity of NH_2_ at C(2) of dihydrotriazine (**5**) could be hampered by the greater steric hindrance exerted by the 4-methoxyphenyl ring. Accordingly, the two magnetically distinguishable protons (δ at ca 6.81 and 5.98 ppm), as imposed by the presence of the 4-methoxyphenyl ring, corresponded to the NH_2_ group at C(2) of dihydrotriazine, while the acetamido nitrogen atom gave rise to a singlet at δ 9.82.

In support of the above NMR characterization of compound **6** structure, according to which the acetyl group is on the 4-amino group of dihydrotriazine ring, we ran the public web FAME 3 (Fast Metabolizer) software [47,48], which allowed us to predict atom positions in compound **5**, as a chemical precursor of **6**, liable to chemical reaction occurring in the live metabolism (i.e., sites of metabolism, SoMs). In particular, we calculated its SoMs probabilities related to phase II reactions, which are characterized by a high predictive performance [47]. Interestingly, the FAME 3 model placed at the top of the generated rank-ordered list of SoMs the NH_2_ at C(4) of dihydrotriazine **5**, recognizing it as the most reactive center to undergo metabolic phase II reaction (probability value of 0.468 over 1), while for the other NH_2_ at C(2) position of **5**, it assigned a two times lower value. Therefore, the outcome of the in silico prediction was in line with the experimental evidence provided by the NMR analysis.

### 2.4. Structure–Activity Relationship Studies of CYC Analogues against Parasitic and Human Enzymes

PYR and the 1-aryl-2,4-diamino-1,6-dihydrotriazines (**1–6**) structurally related to CYC were tested in enzymatic inhibition assays to investigate profiles or their activity against *Tb*PTR1 and *Tb*DHFR-TS in the search for dual targeting anti-*T. brucei* agents. To ascertain a greater selectivity of these molecules for the protozoan enzymes, the study of the human DHFR (hDHFR) inhibition was included. In particular, the chemical variation of the 4-Cl substituent on the phenyl ring and/or of the two methyl groups at C(6) of CYC was explored. The biological results of PYR, CYC, and compounds **1–6** against the investigated targets are depicted in Table 1. The known drug MTX was used as a reference compound because its most important therapeutic action is related to the inhibition of folate-dependent pathways [49].

All compounds were found to target both *Tb*PTR1 and *Tb*DHFR-TS enzymes, even if they showed a preferential selectivity for *Tb*PTR1 (Table 1). The only exception was compound **2**, which exhibited the opposite behaviour, displaying a greater inhibition potency against *Tb*DHFR-TS.

The investigated CYC derivatives proved to be active against *Tb*PTR1, even reaching sub-micromolar potencies in the case of compounds **5** and **6** (IC_50_ = 0.67 µM and 0.83 µM, respectively). The amino groups at C(2) and C(4) of the dihydrotriazine derivatives **1–6** are H-bonded to Ser95 and Tyr174, as in CYC and PYR. Another relevant H-bond involves the NADP(H) phosphate group and the protonated N(3) of dihydrotriazine, being susceptible to protonation in our experimental conditions. Altogether these interactions efficiently stabilize and orient the bioactive conformation of this class of molecules, driving the formation of additional key bonds within the enzyme cavity. Compound **5** differs from CYC by the presence of the polar and electron-donor 4-OCH_3_ group on the phenyl ring in place of the lipophilic and electron-withdrawing 4-Cl atom. This substitution changes the halogen interaction, described for CYC, with the indole of Trp221, involving a stronger H-bond between the NH of Trp221 and the OCH_3_ group, plus van der Waals contacts with the indole ring. The greater energy derived from the contribution of both types of interaction matches with an increased stability of the complex enzyme–inhibitor (**5**), which agrees with the 46-fold improvement of its inhibitory activity against *Tb*PTR1 (IC_50_ = 0.67 µM). The additional introduction of an acetyl group on NH_2_ at C(4) of dihydrotriazine (**5**) still preserves in compound **6** the ability to form H-bonds with Ser95, thus explaining the same degree of potency observed for both these compounds. The 4-OH group of **4** can guarantee the H-bond to the NH of Trp221, but it lacks any van der Waals contacts to the same amino acid residue. As a result, the activity of compound **4** is reduced (IC_50_ = 4.10 µM) with respect to the analogue **5**, but superior to that of CYC.

As next step, a bulky spiro-cyclohexyl moiety was introduced in place of the two methyl groups on C(6) of CYC in order to probe the steric and electronic tolerance of the side chain at the *Tb*PTR1 catalytic site. This structural variation allowed a gain in the inhibitory activity of derivative **3** that demonstrated an IC_50_ value of 1.22 µM, 30-fold lower than CYC. This apolar cycloalkyl group, while colliding with the polar NADP(H) phosphate groups, may ensure extended van der Waals contacts with the hydrophobic pocket lined by Phe97, Val206, Leu209, and Pro210 in *Tb*PTR1. The mono-alkyl substitution at C(6) of CYC (compounds **1** and **2**) also provided activity towards the target enzyme. In particular, the smaller mono-methyl group of **1** may retain a freer conformational geometry than the two more fixed gem-dimethyl groups of CYC, and that enables it to slightly improve the inhibition potency (IC_50_ = 6.02 µM). However, a longer *n*-propyl chain in the same position exerts a negative impact on the activity, which moves back to an IC_50_ value of 27.5 µM, equaling the worst result displayed by the prototype CYC.

Compound **6** showed a similar profile to **5**, the only difference was that the acetyl group on the amine giving the acetamido functionality. Although it was unusual, we suspected the potential hydrolysis of the acetamido group to generate an amine and acetic acid. Therefore, we tested the compound stability at pH 10.5, 7, and 3.7 overnight at room temperature. The HPLC analysis of the incubated solution showed no difference in the chromatograms, suggesting that the compound was fully stable in those conditions. We did not explore the in situ catalysis effect due to the low probability that this event could happen in the enzyme catalytic pocket. Hence, the results suggest that the acetamido group substitution is not affecting the compound affinity and it is not hydrolysed to amine.

### 2.5. Compounds’ Selectivity between the PTR1 and DHFRs Enzymes

Analysis of selectivity profiles (Table 1) revealed the following trends. The selectivity of the drugs PYR, CYC, and MTX was preferential for *Tb*DHFR-TS with respect to *Tb*PTR1, ranging from the lowest value for PYR (selectivity index, SI *Tb*PTR1/*Tb*DHFR-TS = 3.8) up to the highest for CYC (SI *Tb*PTR1/*Tb*DHFR-TS = 123). Therefore, we found that the drug PYR was the best dual targeting agent, showing similar inhibition potencies against both protozoan enzymes. Conversely, CYC analogues were more effective inhibitors of *Tb*PTR1, and the corresponding SI were in the range of 14.4 (**3**)–49.6 (**5**). Compound **2** represented an exception with SI equivalent to that of PYR.

To evaluate the species-selectivity of these molecules, i.e., the preference for the protozoan *Tb*DHFR-TS, they were also tested against hDHFR. In this regard, MTX was also confirmed to block hDHFR activity with the same efficacy observed against *Tb*DHFR-TS, being recognized as potent unselective inhibitor of both DHFRs; this behavior was consistent with its clinical application as an anticancer, anti-inflammatory, and immunosuppressive agent. More interestingly, all other compounds manifested a selectivity index (SI), as the ratio IC_50_ hDHFR/ IC_50_ *Tb*DHFR-TS, more favorable for the protozoan enzyme. In the case of PYR, it was even 20 times higher.

It is worth to note that the chemical variations introduced in CYC analogues **1–6** always yielded a prevailing selectivity for *Tb*DHFR-TS (SI range of 3.2–12.4), thus surpassing that of their prototype (CYC, SI = 1.6). In particular, the best suited chemical modifications for providing a more favorable selectivity towards *Tb*DHFR-TS seemed to be the substitution of 4-Cl on CYC phenyl ring with 4-CH_3_O group (**5**, SI = 12.4), and the presence of a longer and flexible n-propyl chain (**2**, SI = 9.2) in place of the gem-dimethyl the moiety of CYC. Consequently, the combination of all the examined substitutions on the CYC scaffold warrants future investigations. Moreover, the observed poor activity (high micromolar IC_50_s) of CYC analogues against the hDHFR enzyme well matched up with the absence of cytotoxicity we previously reported [38], thus asserting the valuable safety profile of these molecules.

## 3. Discussion

### 3.1. Comparative Analysis of CYC versus PYR TbPTR1 Structure

**Figure 5:**
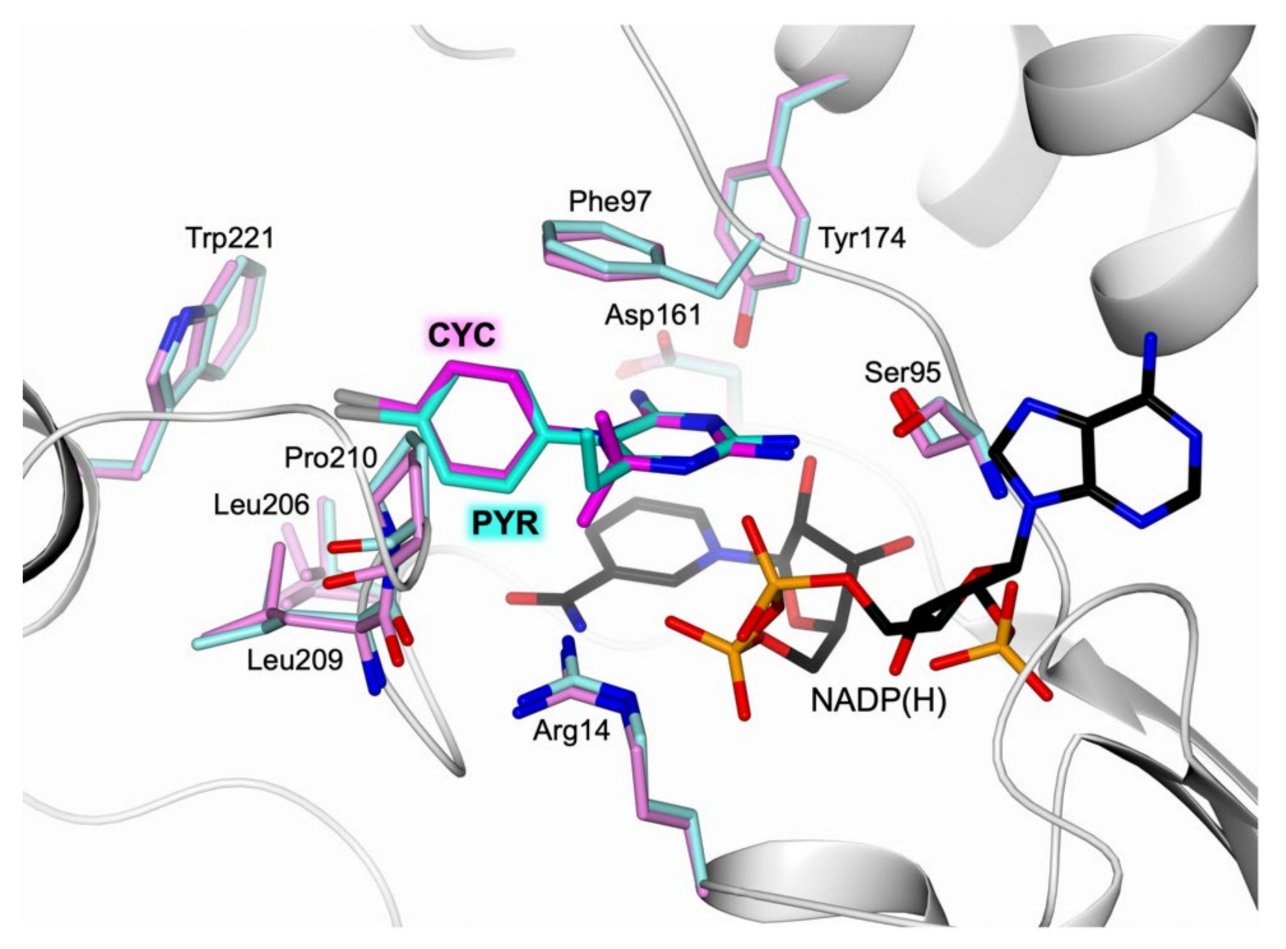
Active-site view of the superimposition between *Tb*PTR1 (white cartoon) in complex with PYR (in sticks, cyan carbon atoms; residues in sticks, light cyan carbons) and CYC (in sticks, magenta carbon atoms; residues in sticks, light magenta carbons; PDB code 6HNC [20]).

PYR and CYC have similar molecular structures, such as a diamine 6-member ring core linked to a 4-chlorophenyl ring (Figure S1, Supplementary Materials). The main difference consists in the chemical structure of the core, i.e., the pyrimidine ring of PYR and the non-aromatic 1,6-dihydrotriazine ring of CYC. They adopt similar binding modes within the *Tb*PTR1 active site, establishing also various common interactions (Figure 5). Altogether, the distorted π-sandwich, the two amine groups, and the nitrogen atoms of PYR and CYC entail the same network of H-bonds with Ser95 and Tyr174 of PTR1 and with the cofactor. Other common interactions involve the 4-chlorophenyl ring of both molecules which establishes the same hydrophobic and halogen bonds with Pro210, Leu209, Val206, Phe97, and Trp221 (Figure 5). Despite these similarities, the inhibition potency of CYC against *Tb*PTR1 is remarkably lower than the one of PYR (IC_50_ of 31.6 µM and 90 nM for CYC [20] and PYR, respectively). The reduced affinity of CYC could be explained by the contribution of some factors that negatively affect the π-sandwich interactions: the presence of a non-aromatic 1,6-dihydrotriazine core with respect to the pyrimidine nucleus of PYR; the reduced conformational flexibility of gem-dimethyl substitution, compared with the mono-ethyl group of PYR, may impose a less favorable bioactive positioning of CYC in the target engagement. Overall, these chemical features may concur to hamper the performance of CYC in the inhibition of *Tb*PTR1.

### 3.2. Structural Basis for Developing Dual Inhibitors of TbPTR1 and TbDHFR

**Figure 6:**
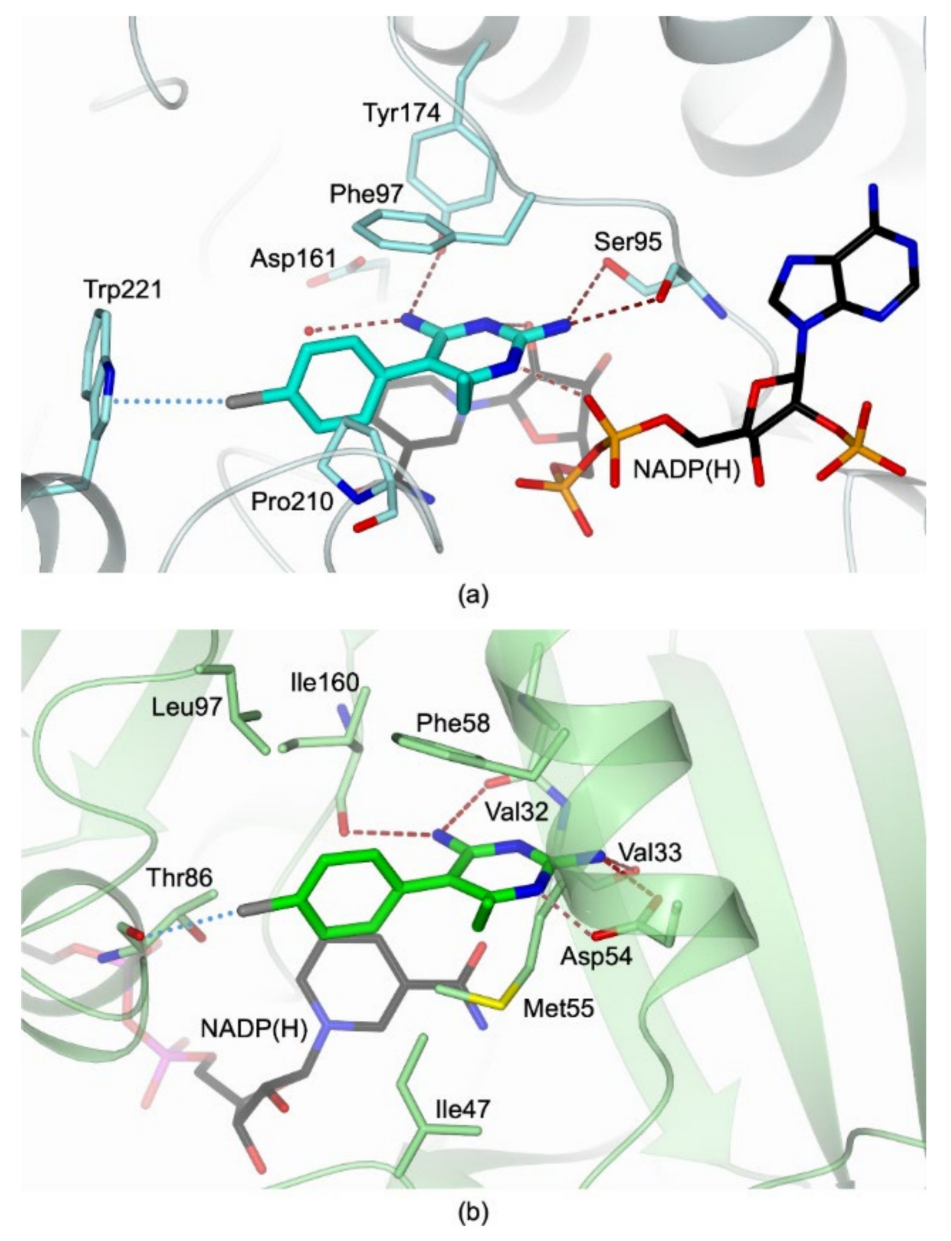
Active-site view of (**a**) TbPTR1 (light cyan cartoon and carbon atoms; residues in sticks) in complex with NADP(H) (in sticks, black carbon atoms) and PYR (in sticks, cyan carbon atoms), (**b**) TbDHFR (light green cartoon and carbon atoms; residues in sticks) in complex with NADP(H) (in sticks, black carbon atoms) and PYR (in sticks, green carbon atoms; (PDB code 3QFX [37]).

As reported above, blocking both DHFR and PTR1 folate-dependent pathways leads to a reduced biosynthesis of nucleic acids and proteins, thus impairing the parasite’s cellular functions [25]. With this purpose, we investigated PYR also as a potential inhibitor of *Tb*PTR1, as its inhibition profile towards *Tb*DHFR was formerly demonstrated (*K_i_* of 24.2 ± 1.3 nM) [37]. Here, we provide evidence that this DHFR inhibitor is also able to effectively target *Tb*PTR1 with an IC_50_ of 90 nM towards the recombinant enzyme. The molecular basis of PTR1 inhibition was elucidated through the determination of the high-resolution structure of the ternary complex *Tb*PTR1:NADP(H):PYR. The comparison with the structure of the ternary complex *Tb*DHFR:NADP(H):PYR (PDB code 3QFX [37]) showed some structural features accounting for the inhibition of both enzymes (Figure 6). Even though DHFR and PTR1 perform analogous catalytic reactions, recognizing and binding the same substrates, their active sites are structurally different and show only a little consensus. Nonetheless, the comparison between the structures of their complexes with PYR led to the identification of the main structural determinants involved in common interactions with both enzymes. In the active site cavities, the 2-amine group of the pyrimidine ring is oriented to entail H-bonds with the surrounding residues of both enzymes, Ser95 in *Tb*PTR1 and Asp54 and Val33 (backbone carbonyl) in *Tb*DHFR. The same binding contribution is also observed for the 4-amine group which forms H-bonds with Tyr174 in *Tb*PTR1 and with Val32 and Ile160 (backbone carbonyls) in *Tb*DHFR (Figure 6). The 6-ethyl group is stabilized by van der Waals contacts with Phe97 and Pro210 in *Tb*PTR1 and with Phe58 and Met55 in *Tb*DHFR. The pyrimidine nitrogen atoms seem not to be fundamental for the inhibitor binding; indeed, differences may be observed between the two enzymes: the N(3) forms H-bonds in both enzymes by interacting with NADP(H) β-phosphate in *Tb*PTR1 and Asp54 carboxylate in *Tb*DHFR; the N(1) is H-bonded to the 4-hydroxyl group of nicotinamide ribose of NADP(H) in *Tb*PTR1 (Figure 6a), whereas it does not take any interaction in *Tb*DHFR (Figure 6b). The size of both catalytic cavities is large enough to allow the rotation of PYR phenyl moiety on the pyrimidine ring, indeed dihedral angles of 74° and 85° have been measured for *Tb*PTR1 and *Tb*DHFR, respectively. Differences in the configuration of the active sites are also evident in the reciprocal orientations of PYR pyrimidine moiety, the phenylalanine ring (Phe97 and Phe58 in *Tb*PTR1 and *Tb*DHFR, respectively), and the cofactor nicotinamide. In *Tb*PTR1, the three aromatic rings form a distorted π-sandwich, a peculiar interaction of this enzyme, ensuring the optimal substrate accommodation to perform the catalytic reaction (Figure 6a). In *Tb*DHFR, a different trend was observed, as the aromatic rings of Phe95 and of the cofactor nicotinamide are not parallel (Figure 6b). Moreover, the reciprocal orientation of phenylalanine and PYR is almost unvaried in the two enzymes, which show the amino acid side chain positioned between the two aromatic rings of the inhibitor entailing hydrophobic interactions. On the opposite side of the *Tb*DHFR cavity, the NADP(H) nicotinamide is proximal to PYR phenyl ring, although these aromatic systems are mutually rotated by ~50°, preventing a π–π interaction. In both enzymes, the PYR 4-chlorophenyl moiety is surrounded by a hydrophobic region lined by Phe58, Ile160, Ile47, and Leu97 in *Tb*DHFR and by Pro210, Leu209, Val206, and Phe97 in *Tb*PTR1. In particular, the 4-Cl atom is engaged in a halogen bond with Trp221 and Thr86 (backbone carbonyl) in *Tb*PTR1 and *Tb*DHFR, respectively.

## 4. Materials and Methods

### 4.1. Synthesis of CYC Analogues

Chemicals and solvents were purchased from Sigma-Aldrich (Milan, Italy). Mps: Büchi apparatus (Milan, Italy), uncorrected. ^1^H NMR spectra and ^13^C NMR spectra were recorded a using a Jeol (Milan, Italy) instrument at 400 and 101 MHz, respectively; chemical shifts were reported as δ (ppm) and referenced to the solvent signal: DMSO-d^6^, quintet at 2.5 ppm (^1^H), septet at 39.5 ppm (^13^C); J in Hz. Elemental analyses were performed on a Flash 2000 CHNS (Thermo Scientific, Milan Italy) instrument in the Microanalysis Laboratory of the Department of Pharmacy, University of Genova. Results of elemental analyses indicated that the purity of all compounds was ≥95%. The dihydrotriazines **1–5** were synthesized according to cited references [43,44,45,46], whereas **6** was synthesized as follows.

Synthesis of the N-[6-amino-5-(4-methoxyphenyl)-4,4-dimethyl-4,5-dihydrotriazin-2-yl]acetamide (**6**).

Compound **5**, obtained as hydrochloride [37], was dissolved in water and precipitated as a free base with an excess of 2N NaOH. The collected 1-(4-methoxyphenyl)-6,6-dimethyl-1,6-dihydro-1,3,5-triazine-2,4-diamine (1.3 mmol) was then reacted at r.t. for 2 h with acetic anhydride (3.9 mmol) in 5 mL of methanol. After evaporation, the oily residue was crystallized with acetone.

Yield: 70%; M.p. 173.5–175.5 °C; ^1^H NMR (400 MHz, DMSO-d^6^): δ 9.82 (s, 1H, CH_3_CONH), 7.22 (d, J = 8.9 Hz, 2H, H arom.), 7.00 (d, J = 9.0 Hz, 2H, H arom.), 6.81 (s, 1H, NH_2_), 5.98 (s, 1H, NH_2_), 3.76 (s, 3H, OCH_3_), 1.60 (s, 3H, COCH_3_), 1.23 (s, 6H, 2CH_3_); ^13^C NMR (101 MHz, DMSO-d^6^) δ 176.67, 160.12, 158.84, 157.99, 131.72 (2C), 128.06, 115.65 (2C), 69.92, 55.91, 27.27 (2C), 25.00. Anal. Calcd for C_14_H_19_N_5_O_2_: C 58.12; H 6.62; N 24.21. Found: C 58.09; H 6.62; N 24.22.

### 4.2. TbPTR1 and TbDHFR-TS Expression, Purification, Kinetics, and Inhibition Assays

Recombinant *Tb*PTR1, *Tb*DHFR-TS, and hDHFR were expressed in *E. coli* as histidine-tagged proteins and purified following established protocols [15]. Purified recombinant enzymes were stored in stock solutions having concentrations of 0.93 mg/mL, 0.53 mg/mL, and 0.88 mg/mL, for *Tb*PTR1, *Tb*DHFR-TS, and hDHFR, respectively. A direct spectrophotometric kinetic test was used to evaluate the inhibitory activity of the compound library. A Jasco-V730 double beam spectrophotometer (Jasco Europe) was employed to perform the assays. Fixed final concentrations of purified *Tb*PTR1, *Tb*DHFR-TS, and hDHFR (15 nM, 55 nM, and 15 nM respectively) were incubated in semi-micro plastic cuvettes (Brand, Tech Scientific, Essex, UK) with 400 µL of sodium citrate buffer at pH = 3.7 and TES buffer (100 mM TES, 50 mM MgCl_2_, 13 mM CH_2_O, 2 mM EDTA, 150 mM 2-mercaptoethanol, pH 7.4), plus 25 µM 7,8-dihydro-L-biopterin (H2B, Sigma Aldrich) for *Tb*PTR1 and 50 µM of 7,8-dihydrofolic acid (H2FA, Sigma Aldrich) for *Tb*DHFR-TS and hDHFR enzymes. After 15 min incubation, 160 µM and 175 µM of buffered NADP(H) coenzyme was pipetted to start the reaction, and the final volume of 800 µL for each cuvette was reached with double deionized water [15].

Inhibitors were dissolved in DMSO to have 100 µM initial stocks, and 1:10 dilutions were performed to minimize the presence of the organic solvent in the reaction cuvette. A DMSO concentration <3% *vol*/*vol* was tested not to interfere with actual reaction rate.

Residual NADP(H) absorption (λ_max_ = 340 nm), converted to NADP^+^ as cofactor, was monitored for 180 s of the ongoing reaction and its Δ(OD)/min was registered for v_(0)_ slope at 22.4 °C. Each inhibition assay was performed in triplicate, and five to six different concentrations of compounds were tested to assess their relative IC_50_. DMSO stocks of 10 µM/1 µM methotrexate (Sigma Aldrich) were used as a positive control for the inhibition of the three enzymes. As the obtained points well fitted a plot with 1/v against the concentrations of compounds, all the investigated molecules were assumed to act as full competitive inhibitor and analysed according to the non-tight binding Michaelis–Menten model.

Reaction rate (as Δ(OD)/min) was plotted against compound concentration, and non-linear regression curves were calculated with the Origin software to obtain the best fitting trend for non-tight binding equilibria. The equation used to interpolate points was y = y_(min)_ + (y_(max)_ − y_(min)_)/(1 + (IC_50_/x)^^HillSlope^), where HillSlope was constrained at −1 (standard Michaelis–Menten behaviour for competitive inhibitors), from which IC_50_ were extrapolated [50]. Standard deviations—as reported in Table 1—were obtained from 95% confidence interval (CI95) of fitting [51].

### 4.3. TbPTR1 and TbDHFR-TS Crystallization

Crystals of *Tb*PTR1 were grown within a few days using the hanging drop vapour diffusion method [52] at room temperature. Well-ordered monoclinic crystals were obtained using a precipitant solution composed of 2–2.5 M sodium acetate and 0.1 M sodium citrate, pH 5, as described elsewhere [15]. The complex with PYR was obtained by the soaking technique, adding 3 mM of the compound (solubilized in DMSO) in crystallization drops containing preformed *Tb*PTR1 crystals (without exceeding a DMSO/crystal solution ratio of 1:9) and incubating them overnight at room temperature. After ~20 h exposure, crystals were transferred to the cryoprotectant solution (obtained by adding 30% v/v glycerol to the precipitant) and then flash frozen in liquid nitrogen.

Crystallization trials were performed also on *Tb*DHFR-TS using the vapour diffusion sitting drop technique either at 8 °C or at room temperature [52]. More than 300 different solutions from Hampton Research (Aliso Viej, CA, USA) and Jena Bioscience (Jena, Germany) crystallization kits (HR2-110/112/144/126/098/211/217, JBSB1-4 and JBSC6 screens) were screened as precipitants. Drops consisting of 1 μL protein solution and 1 μL precipitant were equilibrated against a 200 μL reservoir. No crystal growth was observed over one year of incubation, preventing us from obtaining structural information on this second target.

### 4.4. Data Collection, Structure Solution, and Refinement

Diffraction data were collected at 100 K using synchrotron radiation at the Diamond Light Source (DLS, Didcot, UK) beamline I03 equipped with a Pilatus3 6M detector. Reflections were integrated using XDS [53] and scaled with SCALA [54] from the CCP4 suite [54,55]. The crystals of the complex belonged to the monoclinic space group P2_1_ with a functional tetramer in the asymmetric unit. The structure was solved by the molecular replacement technique as implemented in MOLREP [56] and a functional enzyme tetramer (PDB code 6TBX [21], excluding non-protein atoms and water molecules) was used as searching model. The model was refined using REFMAC5 [57] from the CCP4 suite. The molecular graphic software Coot [58] was used for visual inspection and manual rebuilding of missing atoms in the protein model. The inspection of the Fourier difference map clearly evidenced the presence of the inhibitor inside the catalytic cavity that was modelled and refined in all four subunits of the functional tetramer. The occupancy of exogenous ligands was adjusted and refined to values resulting in atomic displacement parameters close to those of neighbouring protein atoms in fully occupied sites. Water molecules were automatically added using the ARP/wARP suite [59] and verified with Coot [58]. The final model was inspected manually and checked with Coot [58] and PROCHECK [60]. In the final cycles of refinement, all atoms were refined anisotropically (except for those with partial occupancies). Structural figures were generated using the molecular graphic software CCP4mg [61]. Data collection, processing and refinement statistics are summarized in Table S1. Final coordinates and structure factors for the *Tb*PTR1:NADP(H):PYR complex were deposited in Protein Data Bank (PDB) under the code 7OPJ.

### 4.5. Stability of Compound 6 in Solution at Different pH

We have studied the stability of the CYC-like compound **6** to test the hydrolysis of the N-acetyl group in different pH buffers and to screen its putative prodrug action. We prepared three 10 µM solutions from a 100 µM DMSO stock of compound **6** in double deionized water, at pH 10.5 with NaOH, and in citrate buffer, pH 3.7, used for kinetic assays with *Tb*PTR1. We have monitored the purity of these solutions with HPLC–ELSD just after compound dilutions. A volume of 7 µL of each solution was immediately injected in an HPLC Atlantis dC18, 150 × 3.9 mm, 3 µm ID column (Waters Corp, Milford, MA, US) coupled to an ELSD detector (Agilent 1260 Infinity II system). Compounds were eluted with 0.1% formic acid in water (A) and pure acetonitrile (B), gradient 10% to 50% B for 10 min, at 0.5 mL min^−1^, room temperature. Detector parameters were set as follows: Neb = 30 °C, Evap = 40 °C, N_2_ flow = 1.6 SLM, smoothing = 3.0 s. The same amount of compound was injected after 30 h of room temperature incubation, and no changes in chromatograms were detected (Table S2, Figure S2, Supplementary Materials). Compound stability also was evaluated with 1H-NMR, obtaining the same results. We thus concluded that compound **6** is not susceptible to pH mediated hydrolysis, and acts as a *Tb*PTR1 inhibitor as N-acetylated compounds.

## 5. Conclusions

HAT is a severe infectious disease, caused by the protozoan parasite *T. brucei* [1,2]. Currently available treatments against this disease are not satisfactory; therefore, development of new drugs is urgently needed [5]. In trypanosomes, DHFR-TS and PTR1 enzymes play a key role in the production of reduced folates, substances fundamental for the biosynthesis of nucleic acids and proteins. Stating their pivotal role in parasite survival, DHFR and PTR1 are important targets for the development of effective antiparasitic treatments. The identification of molecules able to concomitantly block the activity of both enzymes, leading to parasite cell death, represents an efficient strategy to fight HAT. We formerly showed the ability of CYC to target both *Tb*DHFR and *Tb*PTR1, although reporting a reduced affinity for the latter enzyme. In this investigation, we explored a small library of CYC analogues, **1–6**, having a modified substitution pattern. The comparative analysis of the effects of these modifications on the inhibition of both *Tb*PTR1 and *Tb*DHFR allowed a deeper understanding of the key molecular functions accounting for the inhibition of both enzymes; these findings will guide the development of a next generation of more effective CYC-like dihydrotriazines, as well as examination of the combination of the most relevant chemical features, raising by the present study. To further proceed in the identification of dual inhibitors, we investigated the known drug PYR, reporting an effective inhibition of both enzymes with IC_50_s in the nanomolar range (IC_50_ *Tb*PTR1 = 90 nM, and IC_50_ *Tb*DHFR = 45 nM). The characterization of the ternary complex *Tb*PTR1:NADP(H):PYR provided a clear structural basis to explain its potency towards this second target enzyme. The structural comparison between the binding modes of PYR and CYC in *Tb*PTR1 and *Tb*DHFR has led to obtain key insights for the future design of most promising dual inhibitors exploitable for the treatment of HAT.

## Figures and Tables

**Table 1:** Data on inhibition of *Tb*PTR1, *Tb*DHFR-TS, and hDHFR by pyrimethamine (PYR), cycloguanil (CYC) methotrexate (MTX) and compounds 1-6 and selectivity index all compounds for IC_50_ obtained against *Tb*DHFR-TS *vs Tb*PTR1 and hDHFR *vs Tb*DHFR-TS.

Compound Code	*Tb*PTR1 (IC_50_ µM)	*Tb*DHFR-TS (IC_50_ µM)	hDHFR (IC_50_ µM)	Selectivity IC_50_ *Tb*DHFR-TS /IC_50_ *Tb*PTR1	Selectivity IC_50_ hDHFR /IC_50_ *Tb*DHFR-TS
PYR	0.090	*K_i_* = 0.024 [37]	*K_i_* = 0.47 [38]	c.a. 0.27 ^a^	19.6
CYC	31.6	*K_i_* = 0.256 [37]	*K_i_* = 0.41 [38]	c.a. 0.002 ^a^	1.6
MTX	0.793	0.040	0.014	0.05	0.035
1 (f)	6.02 (±1.10)	103 (±14)	337 (±53)	16.9	3.27
2 (a)	27.5 (±4.54)	7.26 (±2.35)	66.7 (±18.5)	0.26	9.19
3 (b)	1.22 (±0.24)	17.6 (±4.69)	65.6 (±9.3)	14.4	3.73
4 (e)	4.10 (±0.75)	89.0 (±9.9)	284 (±77.4)	21.7	3.19
5 (c)	0.67 (±0.18)	33.2 (±9.64)	413 (±102)	49.6	12.4
6 (d)	0.83 (±0.07)	18.9 (±5.17)	62.5 (±9.1)	22.8	3.31

^a^ In this case the ratio was calculated assuming a competitive inhibition mechanism (see Section 4.2).

## Data Availability

Data is contained within the article and Supplementary Material.

## Supplementary Materials

The following are available online at https://www.mdpi.com/article/10.3390/ph14070636/s1. Figure S1: Chemical structures of MTX, CYC, and PYR; Figure S2: HPLC-ELSD chromatograms of compound 6 after incubation at different pHs; Table S1: Data collection and refinement statistics for the crystal structure of the *Tb*PTR1:NADP(H):PYR complex; Table S2: Calculated %CV for ELDS areas of compound 6.
