# Supplementary material for: Evidence of Pyrimethamine and Cycloguanil Analogues as Dual Inhibitors of Trypanosoma brucei Pteridine Reductase and Dihydrofolate Reductase
Source: Pharmaceuticals (Basel). 2021 Jun 30;14(7):636. doi: 10.3390/ph14070636 (PMC8308740; doi:10.3390/ph14070636)
Supplement: Supplementary file 1 [file pharmaceuticals-14-00636-s001.zip › pharmaceuticals-1263286-supplementary.pdf]

Evidence of pyrimethamine and cycloguanil analogues as dual inhibitors of *Trypanosoma brucei* pteridine reductase and dihydrofolate reductase

Giusy Tassone[1#], Giacomo Landi[1#], Pasquale Linciano[2#], Valeria Francesconi[3], Michele Tonelli[3], Lorenzo Tagliazucchi[2], Maria Paola Costi[2], Stefano Mangani[1], Cecilia Pozzi[1]

[1] Department of Biotechnology, Chemistry and Pharmacy – Department of Excellence 2018-2020, University of Siena, via Aldo Moro 2, 53100 Siena, Italy; giusy.tassone@unisi.it (G.T.); landi31@unisi.it (G.L.): pozzi4@unisi.it (C.P.); stefano.mangani@unisi.it (S.M.)

[2] Department of Life Science, University of Modena and Reggio Emilia, via Campi 103, 41125 Modena, Italy; pasquale.linciano@unipv.it (P.L.); mariapaola.costi@unimore.it (M.P.C.); lorenzo.tagliazucchi@unimore.it (L.T.)

[3] Department of Pharmacy, University of Genoa, viale Benedetto XV n.3, 16132 Genoa, Italy; francesconi.phd@difar.unige.it (V.F.); tonelli@difar.unige.it (M.T.)

Contents

Figure S1 S2

Figure S2 S2

Table S1 S3

Table S2 S4

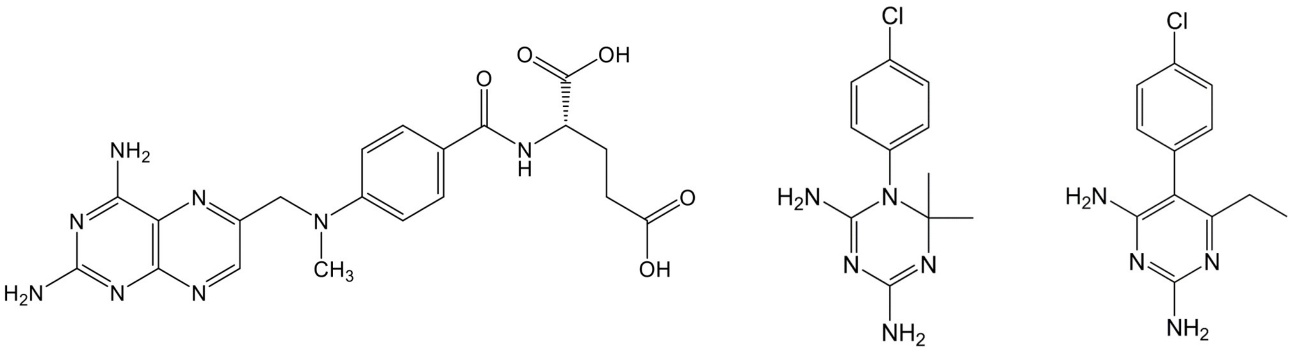

MTX **CYC** **PYR**

Figure S1. Chemical structures of methotrexate (MTX), cycloguanil (CYC), and pyrimethamine (PYR).

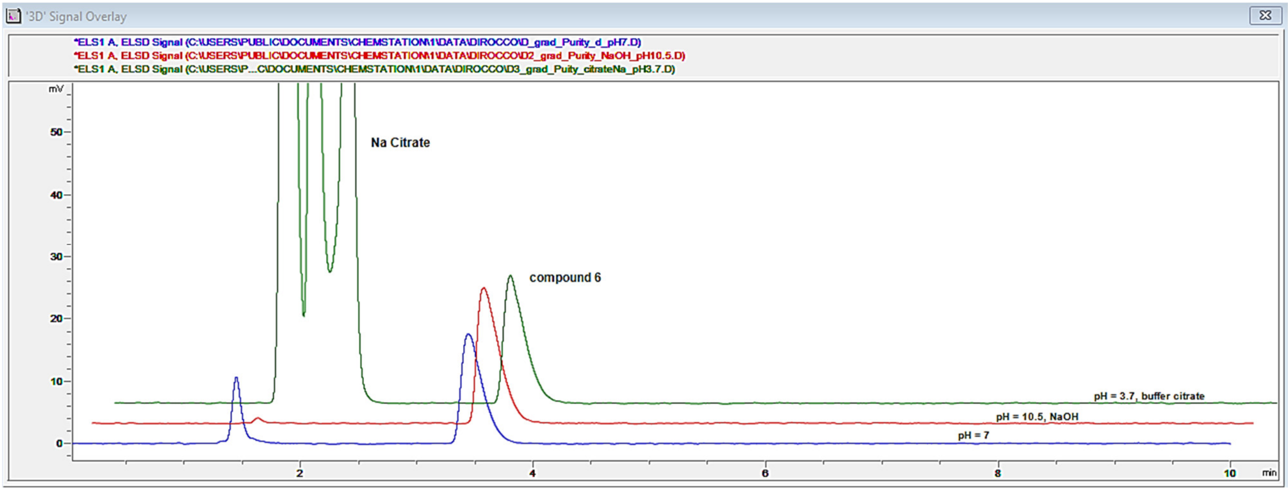

Figure S2. HPLC-ELSD chromatograms of *compound 6* after 30 h incubation in three different pH buffers.

Table S1. Data collection and refinement statistics. Values for the outer shell are given in parentheses.

	*Tb*PTR1:NADP(H):PYR
	**DATA COLLECTION STATISTICS**
PDB ID codes	7OPJ
Diffraction source	I03 (DLS)
Wavelength (Å)	0.91840
Temperature (K)	100
Detector	Pilatus3 6M
Crystal-detector distance (mm)	209.1
Rotation range per image (°)	0.15
Exposure time per image (s)	0.25
Space group	$P2_1$
No. of molecules/ASU	4 (one functional tetramer)
a, *b*, *c* (Å)	74.90, 91.02, 82.80
β (°)	115.63
Resolution range (Å)	18.28 – 1.34 (1.41 – 1.34)
Total no. of reflections	476448 (66781)
No. of unique reflections	210248 (30679)
Completeness (%)	93.9 (94.2)
Redundancy	2.3 (2.2)
$\langle I/\sigma(I)\rangle$	7.6 (2.3)
R_{meas}	0.071 (0.440)
Overall *B* factor from Wilson plot (Å^2)	11.5
	**REFINEMENTS STATISTICS**
Resolution range (Å)	18.07 – 1.34 (1.38 – 1.34)
No. of reflections, working set	199465 (14665)
No. of reflections, test set	10516 (790)
Final R_{cryst}	0.130 (0.231)
Final R_{free}	0.174 (0.275)
No. of non-H atoms	
Protein	7540
PYR	68
NADP(H)	192
Others (acetate, glycerol)	22 (16, 6)
Water	1025
Total	8847
R.m.s. deviations	
Bonds (Å)	0.015
Angles (°)	2.076
Average *B* factors (Å^2)	18.9
Estimate error on coordinates based on R value (Å)	0.049
Ramachandran plot	
Most favored (%)	96.0
Allowed (%)	4.0
RSCC PYR (chain A, B, C, D)	0.96; 0.96; 0.80; 0.96

Table S2. Calculated %CV for ELDS areas of *compound 6* (mV*s).

	Response				Standard deviation	CV %
**SAMPLE**	Area day 1		Area day 2 (after 30h)			
Compound 6_water	297.1	288.6	294.0	279.8	7.58	2.61
Compound 6_CitrateNa	281.7	274.3	288.7	271.3	7.81	2.80
Compound 6_alkaline	291.5	299.5	293.1	297.4	3.71	1.26
